# Prevalence and determinants of frailty among the elderly in the province of Essaouira, Morocco

**DOI:** 10.11604/pamj.2024.48.43.41695

**Published:** 2024-06-05

**Authors:** Hicham Mejdouli, Abdellatif Baali, Hakima Amor, Nadia Ouzennou

**Affiliations:** 1 Laboratory of Pharmacology, Neurobiology, Anthropobiology, Environment and Behaviour, Semlalia Faculty of Sciences, Cadi Ayyad University, Marrakech, Morocco,; 2 Higher Institute of Nursing and Technical Health Professions, Ministry of Health and Social Protection, Marrakech, Morocco

**Keywords:** Frailty, prevalence, elderly, Morocco

## Abstract

**Introduction:**

Morocco is no exception to the global phenomenon of an ageing population. The number of people aged 60 and over rose from one million to 4.5 million between 1970 and 2022. In terms of health, 64.4% of Moroccan seniors are affected by at least one chronic disease, and almost a third suffer from disability. We aimed to estimate the prevalence and identify the factors associated with frailty among the elderly in the Essaouira province of Morocco.

**Methods:**

a descriptive and analytical cross-sectional survey of 384 people aged 65 and over attending health centres in Essaouira province was conducted between March 2022 and January 2023. Data were collected using a self-administered questionnaire. The Fried phenotype was used to assess frailty in the participants.

**Results:**

according to Fried's phenotype, 46.4% of the elderly were frail. Multivariate analyses showed that frailty was associated with family type [OR=1.9; 95% CI 1.4-2.6], professional activity [OR=2.2; 95% CI 1.2-3.9], financial difficulties [OR=1.7; 95% CI 1.1-2.8] and undernutrition [OR=2.9; 95% CI 1.7-4.8].

**Conclusion:**

implementing a screening strategy and speeding up the process of generalising social protection are actions that will make it possible to reduce the prevalence of frailty among the elderly, prevent its complications and act on its main determinants in Morocco.

## Introduction

Population ageing is one of the world's most significant demographic phenomena [1]. It is the result of a combination of lower mortality, lower fertility, and longer life expectancy, often accompanied by a deterioration in health status and poly pathologies [2,3]. Many elderly people experience a transitional period between the diagnosis of chronic illness and the onset of functional dependence. This period is characterized by the onset of frailty, resulting in greater vulnerability and increased susceptibility to the risk of complications [4].

Frailty is presented as a state of unstable equilibrium between robustness and dependence. This intermediate state of equilibrium can be situated more broadly between well-being and illness, the existence of resources and their exhaustion, the presence of an emotional or caring entourage, and total isolation [5]. Fragility can be a physiological precursor and an aetiological factor in disability and dependency [6]. Consequently, the so-called frail person has a higher risk of incapacity or death even in the face of minor external disturbances [7]. It has also been shown that frail people are five times more likely to become dependent, twice as likely to injure themselves, fall, or be hospitalized, and six times more likely to die than non-fragile people, regardless of their social conditions or psychological state [6].

The factors associated with frailty can be classified into four main categories: physiological factors, socio-demographic factors, medical factors and disabilities [8]. However, because it is reversible, identifying frailty is a key factor in replacing the logic of repair with the logic of anticipation [9]. Morocco is no exception to the global phenomenon of ageing [10]. In demographic terms, the number of people aged 60 and over in Morocco rose from one million to 4.5 million between 1970 and 2022. In 2050, it should reach 10 million people, representing a demographic weighting of 23.2% instead of 12.2% at present [11]. In terms of health, the results of the latest National Population and Family Health Survey revealed that 64.4% of elderly people in Morocco are affected by at least one chronic illness [12]. The 2014 National Survey on Disability in Morocco indicated that “*The prevalence rate of incapacity is higher among people aged over 60 (33.7%) due to certain diseases and pathologies that lead to disability and dependency*” [13]. The national survey on mental health indicates that 27.6% of elderly people have a psychological disorder [14]. However, although literature is abundant on the situation of the elderly, frailty in this age group remains partly unexplored by specialist Moroccan researchers. Our aim was therefore to estimate the prevalence and identify the factors associated with frailty among the elderly in the Essaouira province of Morocco.

## Methods

**Type, location and design of the study:** the data in this study are derived from a cross-sectional, descriptive and analytical survey of people aged 65 and over attending health centres in the province of Essaouira between March 2022 and January 2023. The study involved 20 health centres out of 69 urban and rural primary health care facilities. The four urban health centres in the city of Essaouira were included in our sample. Those in rural areas were selected based on one health centre per health district to be representative of the entire province. The province of Essaouira is part of the Marrakech-Safi region, which is located in the centre of Morocco and is one of its 12 regions. Essaouira lies on the shores of the Atlantic Ocean, 174 km west of the city of Marrakech and 173 km north of the city of Agadir. The province of Essaouira has a total population of 449,133, of whom 76.4% live in rural areas. The elderly represent 11.1% of the province's total population.

**Study population:** we recruited 384 people aged 65 and over. Participants were randomly selected during their visits to the health centres until the number of participants per centre was reached.

**Sampling:** proportional stratified random sampling was used to identify the number of participants per centre, based on the number of elderly people served by each facility. This type of analysis made it possible to extract from each stratum representing the elderly, a number in proportion to their numbers in the target population of the twenty health centre study sites. Our sample size was calculated using the Cochran formula:


$$n=\frac{Z^{2}p\left(1-p\right)}{d^{2}}$$

Where, n: sample size; Z: set at a value of 1.96 for a 95% confidence interval; p = standard deviation (p = 0.5); d: tolerated margin of error (5%); hence n= (1.96)^2^*(0.5)*(1-0.5)/(0.05)^2^= 384

**Data collection:** data were collected using a self-administered questionnaire. The questionnaire (paper format) was completed by the elderly at the health centres in the presence of the interviewer, who made it easier for people with physical, comprehension or writing difficulties to complete the questionnaire. The data collected concerning the socio-demographic characteristics (age, genre, marital status, level of education, etc.), frailty and health status (subjective health, chronic diseases, nutritional status and mental health) of the elderly subjects. Frailty was assessed using the Fried phenotype [6]. This is a scale composed of 5 criteria: involuntary weight loss of more than 4.5 kg (or ≥ 5% of body weight) for 1 year, exhaustion felt by the subject, slower walking speed, reduced muscle strength and sedentary lifestyle. Subjects are said to be frail when 3 or more criteria are met. They are said to be “pre-frail” if at least one of the criteria is present. If none of the criteria is present, they are considered robust. We used the short version of the Geriatric Depression Scale (GDS-15) [15]. to study the mental health of the elderly. The normal score is less than 5, depressive symptoms are present for a score of 5 or more. Nutritional status was assessed by calculating the Body Mass Index (BMI). In accordance with the criteria of the French National Authority for Health, subjects with a BMI less than or equal to 21.5 kg/m^2^ were considered malnourished [16]. We used a measuring tape to measure height and an electronic scale to measure weight.

**Data analysis:** the data collected were entered and analysed using “SPSS” software, version 20.0 (IBM Statistical Package for the Social Sciences). The Chi-2 test was used to study the association between the frailty of the group studied and the variables retained in the present study. Binary logistic regression was applied to eliminate confounding factors and capture the weight of associated variables. Statistical significance was set at the 5% threshold.

**Ethical considerations:** the study was carried out after obtaining the necessary authorisations from the health authorities and the ethics committee (Moroccan Association for Research and Ethics, Approval Letter No. 8/REC/21). All participants gave their consent to take part in the study and the interviews were conducted after the care or services for which the elderly people visited the health centres.

## Results

**Socio-demographic and health profile of the elderly people studied:** the socio-demographic, socio-economic, and health characteristics of the subjects studied are given in Table 1. Our sample comprised a total of 384 people aged 65 and over living at home, including 191 men (49.7%) and 193 women (50.3%). The subjects' ages ranged from 65 to 95, with an average of 73.2 years (standard deviation= 7.1 years). The 65-74 age group was the most represented, with a proportion of 64.3%. More than half (53.9%) of the subjects lived in rural areas and 46.1% in urban areas. Concerning marital status, 64.8% of participants were living with a partner and 35.2% were unmarried at the time of the survey. As for educational level, 85.2% were illiterate, 8.6% had primary education and only 6.3% had secondary education or more. Of the 384 elderly people surveyed, one hundred and fifty-seven (40.9%) had a positive perception of old age, 21.9% had a negative perception and 37.2% were indifferent. Most (77.3%) of the subjects interviewed lived with their family, including 62.5% with a large family. Concerning socio-professional activity, 75.3% of participants had no occupation at the time of the survey, and more than half (56.5%) were suffering from financial difficulties. Of the 384 subjects surveyed, 76.3% had basic medical coverage, while 23.7% had no health insurance at all.

**Table 1:** socio-demographic and health characteristics of the subjects studied

Variables	Category	n	%
**Genre**	Men	191	49.7
	Women	193	50.3
**Age groups**	65-74 years	247	64.3
	75-84 years	93	24.2
	≥85 years	44	11.5
**Place of residence**	Urban	177	46.1
	Rural	207	53.9
**Marital status**	Married	249	64.8
	Unmarried	135	35.2
**Level of education**	Illiterate	327	85.2
	Primary	33	8.6
	Secondary and above	24	6.3
**Perception of ageing**	Positive	157	75.3
	Negative	84	24.7
	Neutral	143	40.9
**Type of family**	Solitaire	87	21.9
	Nuclear family	57	37.2
	Large family	240	22.7
**Socio-professional activity**	No profession	289	14.8
	Assets	95	62.5
**Financial difficulties**	Yes	217	56.5
	No	167	43.5
**Medical cover**	Yes	293	76.3
	No	91	23.7
**Subjective state of health**	Good	266	69.3
	Bad	118	30.7
**Chronic illness**	Yes	235	61.2
	No	149	38.8
**Comorbidity**	Yes	101	26.3
	No	283	73.7
**Incapacity**	Yes	205	53.4
	No	179	46.6
**Depressive symptoms**	Yes	141	36.7
	No	243	63.3
**Sleep disorders**	Yes	139	36.2
	No	245	63.8
**Undernutrition**	Yes	146	38.0
	No	238	62.0

n: number; %: percentage

In terms of subjective health, 69.3% of participants felt in good health, including 4% in very good health, while 30.7% felt in poor health, including 3.4% in very poor health. In terms of morbidity, 61.2% of subjects had at least one chronic illness, with 26.3% of participants having a co-morbidity. Hypertension and diabetes were the most prevalent chronic conditions, with prevalence rates of 36.6% and 28.4% respectively. The majority of elderly people (53.4%), i.e. 205 participants, had at least one incapacity. Physical disability was identified in 14.5% of participants, while visual and hearing disabilities were identified in 42.2% and 23.5% of elderly subjects respectively. Depressive symptoms were identified in 36.7% of participants and 36.2% suffered from sleep disorders. The prevalence of undernutrition, as measured by BMI, was 38%.

**Prevalence of frailty:** according to Fried's phenotype, 46.4% of elderly people surveyed (n=178) were frail, 36.2% (n=139) were pre-frail and only 17.4% (n=67) were robust. Table 2 shows the distribution of participants' responses to the various criteria on the frailty scale. Except for the first criterion relating to “involuntary weight loss”, the majority of subjects surveyed had an unfavourable profile in the other 4 criteria. Involuntary weight loss was identified in only 19.5% of participants, while exhaustion was felt by 68.2% of participants, reduced muscle strength was common in 60.2%, slower walking speed was identified in 60.4% and sedentary lifestyles were a feature of daily life in 62.2% of elderly people.

**Table 2:** distribution of participants' responses to Fried's phenotype

Criteria	Yes		No	
	n	%	n	%
**Involuntary weight loss**	75	19.5	309	80.5
**Exhaustion felt by the patient**	262	68.2	122	31.8
**Reduced muscle strength**	231	60.2	153	39.8
**Reduced running speed**	232	60.4	152	39.6
**Sedentary life>**	239	62.2	145	37.8

n: number; %: percentage

**Factors associated with frailty:** the results of the relationship between frailty and the socio-demographic and health characteristics of the subjects studied are presented in Table 3. These results show that frailty is associated, in order of importance, with nutritional status, family type, mental health, age, comorbidity, incapacity, financial difficulties, level of education, sex, socio-professional activity, and subjective health. Multivariate logistic regression analyses (Table 4) show that frailty in our population was statistically associated with family type (OR=1.9; 95% CI 1.4-2.6), socio-professional activity (OR=2.2; 95% CI 1.2-3.9), financial difficulties (OR=1.7; 95% CI 1.1-2.8) and undernutrition (OR=2.9; 95% CI 1.7-4.8).

**Table 3:** frailty and socio-demographic and health characteristics of the subjects studied

Variables	Category	Total (n)	Frailty (%)			P-value
			Normal	Pre-frail	Frail	
**Genre**	Men	191	22.0	40.3	37.7	**0.002**
	Women	193	13.0	32.1	54.9	
**Age groups**	65-74 years	247	20.2	42.1	37.7	**0.000**
	75-84 years	93	15.1	29.0	55,9	
	≥85 years	44	6.8	18.2	75.0	
**Place of residence**	Urban	177	16.4	35.6	48.0	0.801
	Rural	207	18.4	36.7	44.9	
**Marital status**	Married	249	19.3	37.3	43.4	0.226
	Unmarried	135	14.1	34.1	51.9	
**Level of education**	Illiterate	327	15.9	33.9	50.2	**0.006**
	Primary	33	27.3	42.4	30.3	
	Secondary and above	24	25.0	58.3	16.7	
**Perception of ageing**	Positive	157	16.6	38.2	45.2	0.597
	Negative	84	16.7	29.8	53.6	
	Neutral	143	18.9	37.8	43.4	
**Type of family**	Solitaire	87	10.3	13.8	75.9	**0.000**
	Nuclear family	57	15.8	36.8	47.4	
	Large family	240	20.4	44.2	35.4	
**Socio-professional activity**	No profession	289	15.6	34.3	50.2	**0.027**
	Assets	95	23.2	42.1	34.7	
**Financial difficulties**	Yes	217	15.2	29.5	55.3	**0.000**
	No	167	20.4	44.9	34.7	
**Medical cover**	Yes	293	17.7	36.5	45.7	0.904
	No	91	16.5	35.2	48.4	
**Subjective state of health**	Good	266	18.4	39.5	42.1	**0.041**
	Bad	118	15.3	28.8	55.9	
**Chronic illness**	Yes	235	15.7	33.6	50.6	0.104
	No	149	20.1	40.3	39.6	
**Comorbidity**	Yes	101	8.9	27.7	63.4	**0.000**
	No	283	20.5	39.2	40.3	
**Incapacity**	Yes	205	10.2	41.5	48.3	**0,000**
	No	179	25.7	30.2	44.1	
**Depressive symptoms**	Yes	141	3.5	36.2	60.3	**0.000**
	No	243	25.5	36.2	38.3	
**Sleep disorders**	Yes	139	19.4	35.3	45.3	0.744
	No	245	16.3	36.7	46.9	
**Undernutrition**	Yes	146	2.1	36.3	61.6	**0.000**
	No	238	26.9	36.1	37.0	

P-value: Chi-2 test; significance level 5%

**Table 4:** multivariate analysis of socio-demographic and health factors associated with frailty

Variables	Constant	P-value	OR	95%CI	
**Genre**	-0.213	0.398	0.808	0.493	1.325
**Age groups**	0.317	0.124	1.373	0.916	2.058
**Level of education**	0.313	0.234	1.368	0.816	2.293
**Type of family**	0.657	**0.000**	1.929	1.417	2.628
**Socio-professional activity**	0.780	**0.010**	2.182	1.206	3.949
**Financial difficulties**	0.542	**0.031**	1.719	1.050	2.814
**Subjective state of health**	0.003	0.982	1.003	0.759	1.326
**Comorbidity**	0.461	0.133	1.586	0.868	2.898
**Incapacity**	-0.007	0.976	0.993	0.619	1.593
**Depressive symptoms**	-0.442	0.087	0.643	0.388	1.066
**Undernutrition**	1.059	**0.000**	2.884	1.751	4.751

OR: odds ratio; CI: confidence interval

## Discussion

Demographic projections for Morocco suggest that the ageing process will continue over the coming decades. This demographic transition is accompanied by an epidemiological transition characterized by an increase in the prevalence of chronic diseases and disabilities among the elderly. As a result, early detection of vulnerability and frailty is a way of moving from a logic of reparation to one of prevention and anticipation [9]. This study aimed to estimate the prevalence and identify the factors associated with frailty among the elderly in the Essaouira province of Morocco. To achieve this objective, we surveyed a sample of 384 people aged 65 and over, 49.7% of whom were male and 53.9% lived in rural areas.

Our results show that 61.2% of people surveyed had at least one chronic illness, 26.3% of which were co-morbid. Hypertension and diabetes were the most prevalent chronic diseases, with prevalence rates of 36.6% and 28.4% respectively. These results are similar to those of the national survey on population and family health, which indicated that 64.4% of elderly people in Morocco suffer from at least one chronic disease and that hypertension and diabetes are the leading chronic diseases affecting elderly subjects, with proportions of 34% and 20% respectively [12]. Concerning incapacity, our results are in line with those of previous studies which have shown that 12.7% of Moroccan elders suffer from a physical incapacity, 41% have a visual impairment and 24% have a hearing impairment [12,17]. In our sample, physical incapacity was found in 14.5% of the subjects surveyed, visual impairment in 42.2%, and hearing impairment in 23.5%.

Concerning the prevalence of frailty, this study showed that 46.4% of elderly people surveyed were frail. The literature indicates that the prevalence of frailty among the elderly varies from 5 to 58% [18]. In Europe, according to the SHARE study, the proportion of frail elderly people in France is 15% and 27.3% in Italy [19]. In Canada, a 10-year prospective cohort study found that the prevalence of frailty in the elderly was 22.7% [20]. In China, a systematic review of 14 studies conducted in different regions of the country found that the prevalence of frailty in the elderly ranged from 5.9% to 17.4% [21]. In African countries, the prevalence of frailty in the elderly appears to be higher, reaching 35.7% in Cameroon [22], 38% in South Africa and 37.9% in Ghana [23]. According to the French Society of Geriatrics and Gerontology, the clinical expression of frailty is modulated by co-morbidities and by psychological, socioeconomic, and behavioural factors [24]. This variation between studies is therefore explained, of course, by the health characteristics and socio-economic conditions of each population, but also by the differences in the tools used in each study [18].

Most previous studies indicate that frailty increases with age [18,21] and more often affects women [19,25,26]. In our study, bivariate analysis showed that the proportion of frail women (54.9%) exceeded that of men (37.7%), with a statistically significant difference (p<0.01), and that the prevalence of frailty increased significantly with age, reaching 75% in the 85+ age group, compared with only 37.7% in the 65-74 age group (p<0.001). The bivariate analysis also showed that frailty was statistically associated with education level, family type, socio-professional activity, financial difficulties, subjective health, co-morbidity, disability, mental health, and nutritional status. However, binary logistic regression analysis showed that only the variables 'family type', 'socio-professional activity', 'financial difficulties', and 'undernutrition' were significantly and independently associated with frailty.

Concerning family type, this study revealed that the prevalence of frailty rose from 35.4% among subjects living with a large family to 75.9% among those living alone. In Morocco, and the absence of medico-social establishments for housing and caring for the elderly, the family is undoubtedly the most important social institution that meets the needs of the elderly, and the family environment has positive repercussions on the lifestyle, state of health, and quality of life of this category [27]. Moreover, it has been reported that social isolation is associated with frailty [9], whereas family life fosters social ties, solidarity, and mutual aid between family members, and consequently helps to improve the quality of life of the elderly [28].

The economic situation is another important factor associated with frailty in the elderly. This finding is in line with the position of the French Society of Geriatrics and Gerontology, which considers economic characteristics to be one of the determinants of frailty in the elderly [24]. Similarly, a recent study conducted in 6 countries revealed that lower levels of frailty and disability were observed in people with higher levels of wealth [23]. The subjects with no occupation and those with financial difficulties in our sample were more frail. Working for a stable and sufficient income has a positive influence on an individual's quality of life, enabling them to meet their daily needs and also facilitating access to health and social services [27]. Our study also showed that elderly people suffering from malnutrition were more frail than those with satisfactory nutritional status. Moreover, nutritional status is one of the five criteria used in Fried's phenotype to identify frailty [6]. Similarly, a recent systematic review of the literature including 20 studies concluded that frailty in the elderly is associated with body mass index [29]. Finally, this study is not without its limitations, given that it was conducted in only one region out of 12 in Morocco, and did not include patients hospitalized or living in institutions at the time of the survey.

## Conclusion

This study showed that almost half of the elderly people surveyed are frail, and that frailty is associated with social, economic, and nutritional vulnerability. Implementing a screening strategy for frailty in the elderly would be a way of preventing complications from this public health problem. To act on the factors associated with frailty, we believe it is necessary to speed up the process of generalizing social protection in Morocco, which was launched in 2022 and aims, among other things, to generalize health insurance and retirement benefits for the elderly.

### What is known about this topic

- *Frailty in the elderly is presented as a state of unstable equilibrium between robustness and dependence;*
- *Early detection of frailty is a key factor in replacing the logic of repair with a logic of anticipation*.

### What this study adds

- *The prevalence of frailty among the elderly in the Essaouira province of Morocco is very high;*
- *Socio-economic vulnerability is the main determinant of frailty among the elderly in the Essaouira province of Morocco;*
- *The frailty of the elderly is associated with their nutritional status*.
